# The effects of coenzyme Q10 supplementation on lipid profiles among patients with coronary artery disease: a systematic review and meta-analysis of randomized controlled trials

**DOI:** 10.1186/s12944-018-0876-4

**Published:** 2018-10-09

**Authors:** Mohammad Vahid Jorat, Reza Tabrizi, Naghmeh Mirhosseini, Kamran B. Lankarani, Maryam Akbari, Seyed Taghi Heydari, Reza Mottaghi, Zatollah Asemi

**Affiliations:** 1 Cardiovascular Research Center, Shiraz University of Medical Sciences, Shiraz, Iran; 2 Health Policy Research Center, Institute of Health, Student Research Committee, Shiraz University of Medical Sciences, Shiraz, Iran; 3 School of Public Health, University of Saskatchewan, Saskatoon, SK Canada; 4 Health Policy Research Center, Institute of Health, Shiraz University of Medical Sciences, Shiraz, Iran; 5 Research Center for Biochemistry and Nutrition in Metabolic Diseases, Kashan University of Medical Sciences, Kashan, Iran

**Keywords:** Coenzyme Q10, Lipid profiles, Coronary artery disease, Meta-analysis

## Abstract

**Background:**

Chronic inflammation and increased oxidative stress significantly contribute in developing coronary artery disease (CAD). Hence, antioxidant supplementation might be an appropriate approach to decrease the incidence of CAD. This systematic review and meta-analysis was aimed to determine the effects of coenzyme Q10 (CoQ10) supplementation on lipid profile, as one of the major triggers for CAD, among patients diagnosed with coronary artery disease.

**Methods:**

EMBASE, Scopus, PubMed, Cochrane Library, and Web of Science were searched for studies prior to May 20th, 2018. Cochrane Collaboration risk of bias tool was applied to assess the methodological quality of included trials. I-square and Q-tests were used to measure the existing heterogeneity across included studies. Considering heterogeneity among studies, fixed- or random-effect models were applied to pool standardized mean differences (SMD) as overall effect size.

**Results:**

A total of eight trials (267 participants in the intervention group and 259 in placebo group) were included in the current meta-analysis. The findings showed that taking CoQ10 by patients with CAD significantly decreased total-cholesterol (SMD -1.07; 95% CI, − 1.94, − 0.21, *P* = 0.01) and increased HDL-cholesterol levels (SMD 1.30; 95% CI, 0.20, 2.41, *P* = 0.02). We found no significant effects of CoQ10 supplementation on LDL-cholesterol (SMD -0.37; 95% CI, − 0.87, 0.13, *P* = 0.14), lipoprotein (a) [Lp(a)] levels (SMD -1.12; 95% CI, − 2.84, 0.61, *P* = 0.20) and triglycerides levels (SMD 0.01; 95% CI, − 0.22, 0.24, *P* = 0.94).

**Conclusions:**

This meta-analysis demonstrated the promising effects of CoQ10 supplementation on lowering lipid levels among patients with CAD, though it did not affect triglycerides, LDL-cholesterol and Lp(a) levels.

## Background

Dyslipidemia is one of the major risk factor for establishing coronary artery disease (CAD) [1]. Coronary artery disease and cerebral stroke account for the main causes of morbidity and mortality among elderly and middle-aged individuals [2]. Cardiovascular risk factors are including aging, hyperglycemia and insulin resistance [3]. In addition, dyslipidemia, mainly hypercholesterolemia, high LDL-cholesterol, and low HDL-cholesterol levels [4] impair mitochondrial function, leading to increased production of free radicals and reactive oxygen species, and subsequently can result in chronic inflammation and endothelial dysfunction [5].

The damage of free radicals is proposed to play an important role in endothelial dysfunction and atherogenesis [6]. Coenzyme Q10 (CoQ10) is an intracellular antioxidant which prevents senescence and dysfunction caused by oxidative stress [6]. CoQ10 is commonly used to treat cardiomyopathy, and heart function has been remarkably improved following CoQ10 supplementation [7]. CoQ10 deficiency which usually occurs with aging have been shown to increase the risk of type 2 diabetes mellitus (T2DM) [8] and cardiovascular disease (CVD) [9]. On the other hand, there are trials evaluating the effects of CoQ10 on lipid profiles with inconclusive results. We have previously shown in a meta-analysis that taking CoQ10 by patients with metabolic disorders significantly reduced serum triglycerides levels, yet did not affect other lipid profiles [10]. In another meta-analysis conducted by Sahebkar et al. [11], CoQ10 supplementation significantly decreased lipoprotein (a) [Lp(a)] levels among patients with obesity, T2DM, and CVD, mainly in those with Lp(a) ≥ 30 mg/dL. However, in another meta-analysis conducted by Suksomboon et al. [12], CoQ10 supplementation had no beneficial effects on lipid profiles or blood pressures among diabetic patients.

Differences in study design, study population’s characteristics, the dosage of CoQ10 used and the duration of intervention might explain the discrepancies among current evidence. We are aware of no systematic review and meta-analysis of randomized controlled trials (RCTs) on the effect of CoQ10 supplementation on lipid profiles in patients with CAD. This meta-analysis was conducted to summarize the existing evidence of RCTs to evaluate the impact of CoQ10 supplementation on lipid profiles in patients with CAD.

## Methods

PRISMA guideline (the preferred reporting items for systematic reviews and meta-analyses) was used to design and implement this meta-analysis.

### Search strategy

Two independent authors (RT and MA) systematically searched online database including EMBASE, Scopus, PubMed, Cochrane Library, and Web of Science until 20th May 2018. Search words, including Mesh and text words, were applied as followed; (“chronic heart failure (CHF)” OR “CAD” OR “myocardial ischemia (MI)” OR “heart disease (HD)” OR “heart failure, diastolic” OR “CVD” OR “myocardial infarction (MI)” OR “acute myocardial infarction (AMI)” OR “cardiac disease (CD)” AND “Q10” OR “coenzyme Q10 (CoQ10)” OR “supplementation” OR “intake” AND “total cholesterol (TC)” “triglycerides (TG)” OR “low-density lipoprotein (LDL-cholesterol)” OR “LDL-C” OR “high-density lipoprotein (HDL-cholesterol)” OR “HDL-C” OR “lipoprotein(a) (Lp(a))”) to identify the relevant clinical trials examining the impact of CoQ10 supplementation on lipid profiles. To avoid missing any citation that had not been captured in the primary search, the reference lists of the relevant papers and the pervious article reviews were searched by the same two researchers (RT, MA). With no time restriction, clinical trials published in English were included in this meta-analysis.

### Study selection

Two investigators (RT, MA) independently screened all studies, retrieved from the online database and hand-search, using a two-stage process in order to determine eligible studies for current meta-analysis. In the first stage, after reviewing the title and/or abstracts of the trials, any duplicate and non-relevant trial was removed from further process. Then, in the next stage, the full texts of related trials were retrieved to determine whether trials were potentially ideal for this meta-analysis, considering inclusion and exclusion criteria. Upon any disagreement, it was resolved by a discussion among themselves or with a third investigator (ZA or MV.J).

The following inclusion criteria was used to select eligible clinical trials for the meta-analysis: 1) being a human original RCT with parallel or crossover method; 2) study participants were patients diagnosed with heart diseases; 3) being a placebo-controlled clinical trial assessing the association between CoQ10 supplementation and lipid profiles; 4) clinical trials that reported the mean changes (SD or 95% CI) of the impact of CoQ10 on lipid profiles including total- and LDL-cholesterol, triglycerides, HDL-cholesterol, and Lp(a) at baseline and end of intervention in both intervention and placebo groups.

Animal or in-vitro trials, clinical trials without control groups, no RCT design, and any trial protocols, the congress abstracts without full text, and the studies did not meet the minimum requirement for quality assessment score were excluded from this meta-analysis.

### Data extraction and quality assessment

The quality assessment and data extraction from each included trial were conducted by two independent investigators (RT and MA), using Cochrane Collaboration risk of bias tool and standard Excel sheet-form 2008, respectively. Any disagreement was discussed by a third investigator (ZA or MV.J).

The following study characteristics were used to assess the quality of included trials: “randomization generation, allocation concealment, blinding of participants and outcome assessors, incomplete outcome data, selective outcome reporting, and other sources of bias”. The following data was extracted from each clinical trial: first author’s name, year of publication, mean age, country of origin, study design, sample size (in intervention and placebo groups), duration of trial, dose of supplement, type of treatment and placebo in both intervention and control groups, the mean and SD/or 95% CI for total- and LDL-cholesterol, triglycerides, HDL-cholesterol, lipoprotein (a) concentrations at baseline and end of intervention in both intervention groups.

### Statistical analyses

All statistical analyses were conducted using STATA version 12.0 (Stata Corp, College Station, TX) and Review Manager V.5.3 software (Cochrane Collaboration, Oxford, UK). The Cochran (Q) and I-squared statistics (I^2^) were applied to show the heterogeneity among included trials. Upon existing heterogeneity across trials (I^2^ ≥ 50% with *P* < 0.05), random effects model with DerSimonian and Laird method; otherwise, fixed-effect model with inverse variance method were used to calculate the standardized mean differences (SMDs) with 95% CI considering as the pool effect size. Further, subgroup analyses were used based on pre-defined criteria such as type of disease (HF vs. CAD), type of intervention (Q10 vs. Q10 plus other nutrients), dosage of supplement (≤ 150 vs. > 150 mg/day), duration of trial (< 8 weeks vs. ≥8 weeks), and sample size (≤50 vs. > 50 individuals in intervention groups) to identify the source of heterogeneity based on confounding variables.

Sensitivity analyses were conducted to specify the effect of each included trial on the pooled SMDs, using leave-one-out method. Egger’s and Begg’s tests were used to indicate the potential publication bias for the outcome measures. *P* < 0.05 was considered as statistically significant.

## Results

**Fig. 1:**
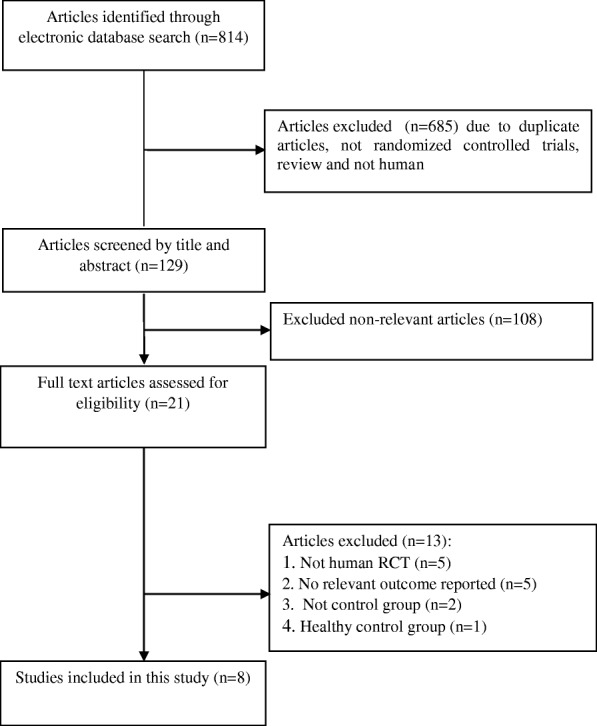
Literature search and review flowchart for selection of studies

**Fig. 2:**
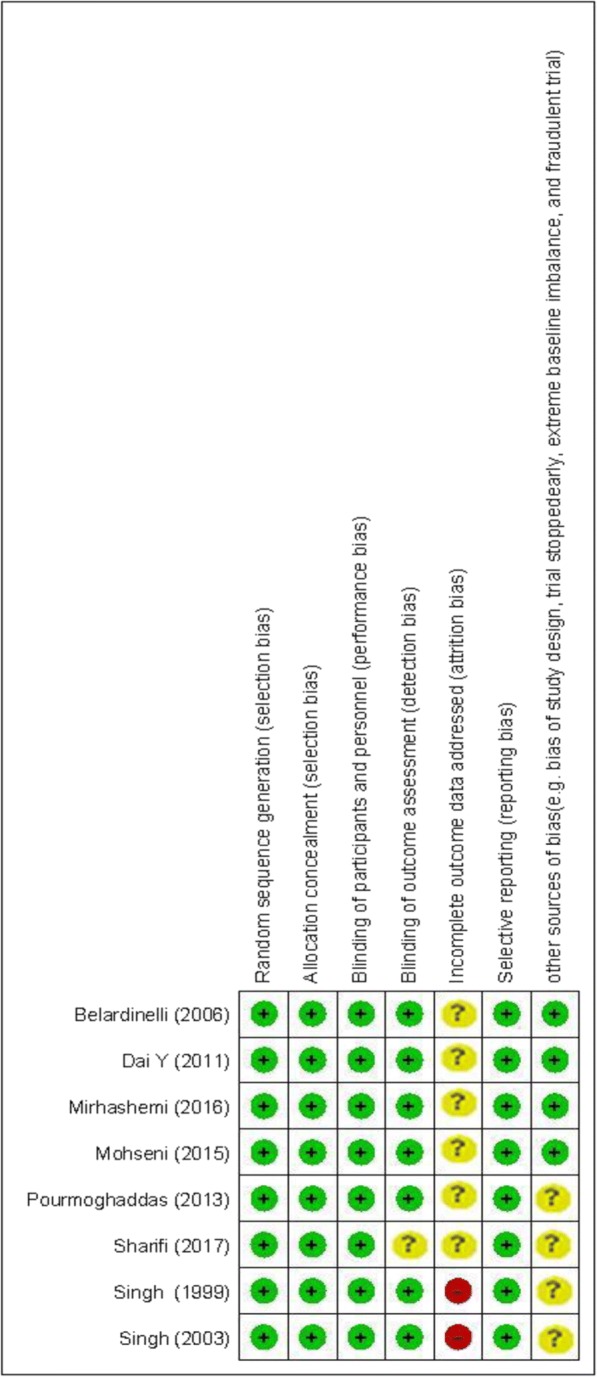
The summary of review authors’ judgments about each risk of bias item for each included study

A total of 814 studies were identified though our initial literatures search. After screening RCTs, 8 studies were determined to be appropriate for in the present meta-analysis. The flow diagram of step by step studies identification and selection has been illustrated in Fig. 1. Seven clinical trials had double-blinded design and one of the trials was single-blinded. Seven trials were conducted using parallel design and one cross-over design. Eight RCTs reported the effects of CoQ10 supplementation on total cholesterol, 6 on LDL-cholesterol, 4 on triglycerides, 5 on HDL-cholesterol, and 3 on Lp(a). Sample size varied from 21 to 73 participants with an overall number of 267 subjects. The sample size in the placebo group was ranged from 21 to 71 with an overall number of 259 subjects. Duration of the intervention among included trials was ranged from 4 to 48 weeks. The detailed characteristics of included trials were summarized in Table 1. The assessment of the methodological quality of the included trials has been indicated in Fig. 2. The findings of risk of bias assessment indicated that 6 studies were at unclear risk of bias, and 2 studies were at high risk bias based on the judgments of author.

**Table 1:** Characteristics of included studies

Authors (Ref)	Publication year	Sample size (control/intervention)	Country/population	Intervention (name and daily dose)	Duration	Age (control, intervention)	Presented data
Belardinelli et al. [23]	2006	21/21	Italy/chronic HF	CoQ10 100 mg/day	4 weeks	60 ± 8	TC, LDL-C, TG, HDL-C
Dai Y et al. [24]	2011	28/28	Hong Kong/left ventricular systolic dysfunction	CoQ10 300 mg/ day	8 weeks	65.9 ± 12.5, 59.9 ± 13.1	TC, LDL-C, HDL-C, Lp(a)
Mohseni et al. [15]	2015	26/26	Iran/acute myocardial infarction	CoQ10 200 mg/day	12 weeks	48.4 ± 0.5, 47.6 ± 0.3	TC, LDL-C, TG, HDL-C
Mirhashemi et al. [25]	2016	30/30	Iran/T2DM with CAD	CoQ10 100 mg/day	8 weeks	68.9 ± 9.6	Lp(a)
Pourmoghaddas M et al. [26]	2013	30/32	Iran/statin treated coronary heart failure patients	CoQ10 200 mg/ day	16 weeks	48.0 ± 8.6, 47.6 ± 8.2	TC, LDL-C
Singh et al. [19]	1999	22/25	India/CAD	CoQ10 120 mg/day	4 weeks	50.70 ± 12.5, 54.47 ± 14.6	TC, LDL-C, HDL-C, Lp(a)
Singh et al. [14]	2003	71/73	India/myocardial infarction	CoQ10 120 mg/day	48 weeks	51 ± 9, 51 ± 10	TC, LDL-C, TG, HDL-C
Sharifi et al. [13]	2017	31/32	Iran/myocardial infarction	CoQ10 150 mg/day and L-carnitine 1200 mg/day	3 months	59 ± 9	TC

*CAD* coronary artery disease, *HDL-C* high density lipoprotein-cholesterol, *HF* heart failure, *LDL-C* low density lipoprotein-cholesterol, *Lp(a)* Lipoprotein(a), *TC* total cholesterol, *TG* triglycerides, *T2DM* type 2 diabetes mellitus

### Effects of CoQ10 on lipid profiles

**Fig. 3:**
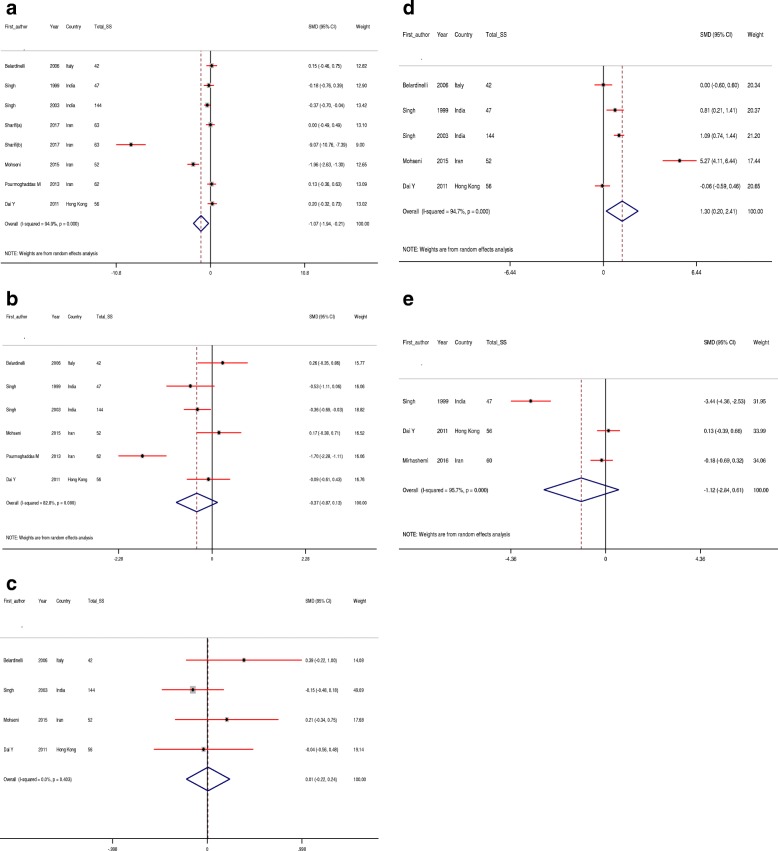
A-E. Meta-analysis lipid profiles standardized mean differences estimates for (**a**) total cholesterol, **b** for LDL-cholesterol, **c** for triglycerides, **d** for HDL-cholesterol and (**e**) for lipoprotein(a) in CoQ10 and control groups (CI = 95%).Total SS, total sample size

Using random-effect model, the pooled results for lipid profiles showed that CoQ10 supplementation significantly decreased total cholesterol (SMD -1.07; 95% CI, − 1.94, − 0.21, *P* = 0.01; I^2^ = 94.9%) and increased HDL-cholesterol levels (SMD 1.30; 95% CI, 0.20, 2.41, *P* = 0.02; I^2^ = 94.7%). We found no significant impact of CoQ10 supplementation on LDL-cholesterol (SMD -0.37; 95% CI, − 0.87, 0.13, *P* = 0.14; I^2^ = 82.8%) and Lp(a) (SMD -1.12; 95% CI, − 2.84, 0.61, *P* = 0.20; I^2^ = 95.7%), using random-effect model and triglycerides levels (SMD 0.01; 95% CI, − 0.22, 0.24, *P* = 0.940; I^2^ = 0.00%), using fixed-effect model (Fig. 3).

The detailed results of meta-analyses for the effects of CoQ10 on lipid profiles including total- and LDL-cholesterol, triglycerides, HDL-cholesterol, lipoprotein(a) concentrations at baseline and end of the trial in both intervention and placebo groups have been presented in Table 2.

**Table 2:** Estimation of the effects of CoQ10 supplementation on lipid profiles at baseline and the end of the treatment between intervention and placebo groups

Variable		Number of study	Standardized Mean difference	CI 95%	*P*-value	Heterogeneity		
						I^2^ (%)	Q	*P*-value Heterogeneity
TC	Intervention group (after vs. before)	7	-1.07	−1.88, − 0.26	0.009	93.7	95.40	< 0.001
	Placebo group (after vs. before)	7	−0.37	− 0.56, − 0.19	< 0.001	42.0	10.34	0.11
	Change intervention group vs. placebo group	8	−1.07	−1.94, − 0.21	0.015	94.9	138.32	< 0.001
LDL-C	Intervention group (after vs. before)	5	−0.71	−1.30, − 0.12	0.019	84.8	26.27	< 0.001
	Placebo group (after vs. before)	5	−0.55	−1.21, 0.10	0.098	87.3	31.60	< 0.001
	Change intervention group vs. placebo group	6	−0.37	−0.87, 0.13	0.149	82.8	29.05	< 0.001
TG	Intervention group (after vs. before)	3	0.15	−0.38, 0.68	0.591	71.6	7.04	0.03
	Placebo group (after vs. before)	3	−0.09	−0.34, 0.17	0.493	0.00	0.31	0.85
	Change intervention group vs. placebo group	4	0.01	−0.22, 0.24	0.940	0.00	2.93	0.40
HDL-C	Intervention group (after vs. before)	4	0.54	−0.07, 1.16	0.081	82.3	16.93	0.001
	Placebo group (after vs. before)	4	−0.01	− 0.24, 0.23	0.964	0.00	0.35	0.95
	Change intervention group vs. placebo group	5	1.30	0.20, 2.41	0.021	94.7	76.05	< 0.001
Lp(a)	Intervention group (after vs. before)	2	− 0.71	− 1.91, 0.50	0.252	89.1	9.19	0.002
	Placebo group (after vs. before)	2	0.02	−0.37, 0.40	0.929	0.00	0.17	0.68
	Change intervention group vs. placebo group	3	−1.12	−2.84, 0.61	0.204	95.7	46.96	< 0.001

*HDL-C* high density lipoprotein-cholesterol, *LDL-C* low density lipoprotein-cholesterol, *Lp(a)* Lipoprotein (a); *TC* total cholesterol, *TG* triglycerides

The findings of sensitivity analyses revealed that pooled SMDs for the impact of CoQ10 on LDL-cholesterol and triglycerides were robust, and removing each trial did not affect the overall results of meta-analysis. Further, the pooled SMDs for total cholesterol was significantly different between the pre- (− 1.07; 95% CI, − 1.94, − 0.21) and post-sensitivity pooled SMD (− 0.26; 95% CI, − 0.72, 0.18) after removing Sharifi et al. [13]. For HDL-cholesterol also, the pre- (1.30; 95% CI, 0.20, 2.41) and post-sensitivity pooled SMD were significantly different after excluding Singh et al. [14] (1.41; 95% CI, − 0.20, 3.03) and Mohseni et al. [15] (0.47; 95% CI, − 0.13, 1.09) (Table 3).

**Table 3:** The assess of contribution each clinical trials in association between CoQ10 supplementation and lipid profiles based on sensitivity analysis

Variable	Pre-sensitivity analysis			Upper & lower of effect size	Post-sensitivity analysis		
	No. of studies included	Pooled SMD (random effect)	95% CI		Pooled SMD (random effect)	95% CI	Excluded studies
TC	8	−1.07	− 1.94, − 0.21	Upper	−0.26	− 0.72, 0.18	Sharifi (b)
				Lower	−1.30	−2.29, −0.31	Dai Y
LDL-C	6	−0.37	−0.87, 0.13	Upper	−0.14	− 0.42, 0.12	Pourmoghaddas
				Lower	−0.48	− 1.04, 0.06	Belardinelli
TG	4	0.01	−0.22, 0.24	Upper	0.16	−0.15, 0.48	Singh (2003)
				Lower	−0.05	− 0.30, 0.19	Belardinelli
HDL-C	5	1.30	0.20, 2.41	Upper	1.67	0.32, 3.03	Dai Y
				Lower	0.47	−0.13, 1.09	Mohseni
Lp(a)	3	−1.12	−2.84, 0.61	Upper	− 0.03	−0.39, 0.33	Singh (1999)
				Lower	−1.79	−4.98, 1.40	Manolescu

*HDL-C* high density lipoprotein-cholesterol, *LDL-C* low density lipoprotein-cholesterol, *Lp(a)* Lipoprotein (a); *TC* total cholesterol, *TG* triglycerides

Considering type of disease, the results of subgroup analysis showed a significant reduction in total cholesterol concentrations among patients with CAD (SMD -2.01; 95% CI, − 3.43, − 0.60) rather than those with HF (SMD 0.16; 95% CI, − 0.15, 0.47). Trials applying dosages ≤150 mg/day indicated greater reduction in total cholesterol levels (SMD -1.53; 95% CI, − 2.83, − 0.23) compared with the dosage of intervention > 150 mg/day (SMD -0.52; 95% CI, − 1.78, 0.73). Moreover, compared with trials shorter than 8 weeks, total cholesterol concentrations showed greater reduction in trials with ≥8 weeks’ supplementation (0.06 vs. -1.94, 95% CI: -3.33, − 0.54) (Table 4). LDL-cholesterol and Lp(a) levels did not show any significant difference applying potential moderators in subgroup analyses (Table 4).

**Table 4:** The effects of CoQ10 supplementation on lipid profiles with CI 95% by using subgroup analysis

Variables	K^a^	I^2^ (%)	Q test	SMD (95% CI)	*P*-value
TC					
Total	8	94.9	138.32	−1.07 (−1.94, −0.21)	0.015
Type of disease					
HF	3	0.00	0.04	0.16 (−0.15, 0.47)	0.306
CAD	5	96.7	122.87	−2.01 (−3.43, −0.60)	0.005
Type of intervention					
Q10	6	84.7	32.72	−0.32 (−0.85, 0.22)	0.244
Q10 + other	2	99.0	102.67	−4.50 (−13.39, 4.39)	0.321
Dosage of intervention (mg/dL)					
≤ 150	5	96.3	107.92	−1.53 (−2.83, −0.23)	0.021
> 150	3	93.4	30.37	−0.52 (−1.78, 0.73)	0.414
Duration of study					
< 8 weeks	3	0.00	1.06	0.06 (−0.26, 0.39)	0.707
≥ 8 weeks	5	96.9	128.76	−1.94 (−3.33, − 0.54)	0.006
Sample size					
≤ 50 person	2	0.00	0.60	−0.03 (− 0.44, 0.39)	0.13
> 50 person	6	96.3	135.05	−1.52 (−2.67, − 0.37)	2.59
LDL-C					
Total	6	82.8	29.05	−0.37 (− 0.87, 0.13)	0.149
Type of disease					
HF	3	91.8	24.47	−0.51 (−1.66, 0.64)	0.387
CAD	3	42.7	3.49	−0.25 (− 0.61, 0.11)	0.167
Type of intervention					
Q10	6	82.8	29.05	−0.37 (− 0.87, 0.13)	0.149
Q10 + other	–	–	–	–	–
Dosage of intervention					
≤ 150	3	48.7	3.90	−0.24 (− 0.64, 0.16)	0.232
> 150	3	91.8	24.25	−0.53 (−1.64, 0.57)	0.344
Duration of study					
< 8 weeks	3	40.5	3.36	−0.12 (− 0.55, 0.30)	0.568
≥ 8 weeks	3	91.2	22.64	−0.62 (−1.55, 0.31)	0.195
Sample size					
≤ 50 person	2	70.0	3.33	−0.14 (− 0.91, 0.63)	0.721
> 50 person	4	87.8	24.52	−0.48 (−1.17, 0.21)	0.171
HDL-C					
Total					
Type of disease	5	94.7	76.05	1.30 (0.20, 2.41)	0.021
HF	2	0.00	0.02	−0.04 (−0.43, 0.36)	0.859
CAD	3	95.9	48.48	2.28 (0.51, 4.05)	0.011
Type of intervention					
Q10	5	94.7	76.05	1.30 (0.20, 2.41)	0.021
Q10 + other	–	–	–	–	–
Dosage of intervention					
≤ 150	3	78.5	9.29	0.66 (0.03, 1.30)	0.041
> 150	2	98.5	66.76	2.58 (−2.65, 7.81)	0.334
Duration of study					
< 8 weeks	3	62.7	5.36	0.24 (−0.30, 0.78)	0.387
≥ 8 weeks	2	97.8	45.29	3.14 (−0.96, 7.24)	0.133
Sample size					
≤ 50 person	2	71.2	3.48	0.41 (−0.39, 1.20)	0.315
> 50 person	3	97.0	67.48	2.00 (0.06, 3.93)	0.043
Lp(a)					
Total	3	95.7	46.96	−1.12 (−2.84, 0.61)	0.204
Type of disease					
HF	1	–	0.00	0.13 (−0.39, 0.66)	0.621
CAD	2	97.3	37.38	−1.79 (−4.98, 1.40)	0.272
Type of intervention					
Q10	3	95.7	46.96	−1.12 (−2.84, 0.61)	0.204
Q10 + other	–	–	–	–	–
Dosage of intervention					
≤ 150	2	97.3	37.38	−1.79 (− 4.98, 1.40)	0.272
> 150	1	–	0.00	0.13 (−0.39, 0.66)	0.621
Duration of study					
< 8 weeks	2	97.7	44.27	−1.64 (−5.14, 1.87)	0.360
≥ 8 weeks	1	–	0.00	−0.18 (− 0.69, 0.32)	0.476
Sample size					
≤ 50 person	1	–	0.00	−3.44 (−4.36, −2.53)	< 0.001
> 50 person	2	0.00	0.72	−0.03 (− 0.40, 0.33)	0.866

^a^ K, Number of SMD included; *CAD* coronary artery disease, *HDL-C* high density lipoprotein-cholesterol, *HF* heart failure, *LDL-C* low density lipoprotein-cholesterol, *Lp(a)* Lipoprotein(a), *TC* total cholesterol

CoQ10 supplementation significantly increased HDL-cholesterol concentrations in patients with CVD (SMD 2.28; 95% CI, 0.51, 4.05) rather than those with HF (SMD -0.04; 95% CI, − 0.43, 0.36). Considering the dosage of intervention, subgroup analysis revealed that CoQ10 doses ≤150 mg/day significantly increased HDL-cholesterol concentrations (SMD 0.66; 95% CI, 0.03, 1.30) rather than doses of > 150 mg/day intervention (SMD 2.58; 95% CI, − 2.65, 7.81). Further, with the total sample size > 50 participants, HDL-cholesterol levels were significantly higher (SMD 2.00; 95% CI, 0.06, 3.93) when compared to trials with ≤50 participants (SMD 0.41; 95% CI, − 0.39, 1.20). The detailed results of subgroup analyses have been summarized in Table 4.

### Publication bias

Begg’s and Egger’s statistics indicated no significant evidence of publication bias for the meta-analyses evaluating the effects of CoQ10 on total cholesterol (Begg’s: Z = − 0.99, *P* = 0.32 and Egger’s: B = − 8.96, *P* = 0.05), LDL-cholesterol (Begg’s: Z = 0.19, *P* = 0.85 and Egger’s: B = − 0.51, *P* = 0.91), triglycerides (Begg’s: Z = 1.07, *P* = 0.08 and Egger’s: B = 2.99, *P* = 0.10), HDL-cholesterol (Begg’s: Z = 0.49, *P* = 0.62 and Egger’s: B = 5.31, *P* = 0.43), and Lp (a) (Begg’s: Z = 0.52, *P* = 0.60 and Egger’s: B = − 7.28, *P* = 0.50).

## Discussion

The findings of current systematic review and meta-analysis showed that CoQ10 supplementation significantly improved lipid profiles by decreasing total cholesterol and increasing HDL-cholesterol levels, though did not affect triglycerides, LDL-cholesterol and Lp(a) levels in patients with CAD.

CoQ10 deficiency usually occurs with aging and may increase the risk of CVD [9, 16, 17]. We have previously demonstrated in another meta-analysis that taking CoQ10 by patients with metabolic disorders significantly reduced serum triglycerides levels, yet did not affect other lipid profiles [10]. Meta-analysis conducted by Pirro et al. [18] showed that taking a nutraceutical combination of red yeast rice, berberine, policosanol, astaxanthin, CoQ10 and folic acid significantly reduced serum triglycerides, total-, LDL-and HDL-cholesterol levels. Supplementation with 200 mg/day CoQ10 for 12 weeks significantly increased HDL-cholesterol levels in hyperlipidemic patients with myocardial infarction, though did not affect other parameters of lipid profiles [15]. Supplementation with 120 mg/day CoQ10 for 28 days led to similar results among patients with acute coronary disease [19]. In a meta-analysis conducted by Suksomboon et al. [12], total cholesterol levels significantly decreased after taking CoQ10 plus fenofibrate. The anti-hyperlipidemic effect of fenofibrate has already been proven; though its synergistic impact with CoQ10 is promising. Significant reduction in serum triglyceride levels was reported following supplementation with CoQ10 plus fenofibrate [12]. However, Sahebkar et al. [11] documented in their meta-analysis that CoQ10 supplementation significantly decreased plasma Lp(a) levels in individuals with lipoprotein a [Lp(a)] ≥30 mg/dL, though other lipid profiles parameters remained unchanged. In addition, CoQ10 administration to diabetic patients had no beneficial effects on lipid profiles and blood pressure [12].

The type and different dosages of CoQ10 used are some of the possible reasons that might explain the discrepant results among previous published studies. Although the main reason CoQ10 affects lipid profiles is unknown; several mechanisms have been proposed by which CoQ10 supplements could improve lipid profiles. As an intracellular antioxidant, CoQ10 protects the cell membrane phospholipids and mitochondrial membrane protein against free radical-induced damage [6]. Aldehyde derivatives from lipid peroxidation, such as malondialdehyde, inhibit lecithin-cholesterol acyl transferase (LCAT), which esterifies free cholesterol on HDL-cholesterol. So, CoQ10 can promote HDL-cholesterol production, suppress oxidative stress and subsequently reduce malondialdehyde levels [20]. In addition, CoQ10 ingestion may induce gene expression of peroxisome proliferator-activated receptor-γ (PPAR-γ) through activating calcium-mediated AMPK pathway and inhibiting differentiation-induced adipogenesis [21]. PPAR-γ is a nuclear receptor protein which acts as a ligand-activated transcription factor in regulating gene expression affecting insulin and lipid metabolism, differentiation, proliferation, survival and inflammation [22].

## Conclusions

CoQ10 supplementation significantly improved some of the parameters of lipid profile including total cholesterol and HDL-cholesterol levels in patients with CAD, though it might not affect the other parameters of lipid profiles. Additional prospective studies regarding the effect of CoQ10 intake on lipid profiles in patients with CAD are necessary.

## Abbreviations

HDL-C: high density lipoprotein-cholesterol
LDL-C: Low density lipoprotein-cholesterol
Lp(a): Lipoprotein(a)
RCTs: Randomized controlled trials
TC: Total cholesterol
TG: Triglycerides
